# Using Rich Data on Comorbidities in Case-Control Study Design with Electronic Health Record Data Improves Control of Confounding in the Detection of Adverse Drug Reactions

**DOI:** 10.1371/journal.pone.0164304

**Published:** 2016-10-07

**Authors:** Daniel Backenroth, Herbert Chase, Carol Friedman, Ying Wei

**Affiliations:** 1 Department of Biostatistics, Columbia University, New York, New York, United States of America; 2 Department of Biomedical Informatics, Columbia University, New York, New York, United States of America; National Chiao Tung University College of Biological Science and Technology, TAIWAN

## Abstract

Recent research has suggested that the case-control study design, unlike the self-controlled study design, performs poorly in controlling confounding in the detection of adverse drug reactions (ADRs) from administrative claims and electronic health record (EHR) data, resulting in biased estimates of the causal effects of drugs on health outcomes of interest (HOI) and inaccurate confidence intervals. Here we show that using rich data on comorbidities and automatic variable selection strategies for selecting confounders can better control confounding within a case-control study design and provide a more solid basis for inference regarding the causal effects of drugs on HOIs. Four HOIs are examined: acute kidney injury, acute liver injury, acute myocardial infarction and gastrointestinal ulcer hospitalization. For each of these HOIs we use a previously published reference set of positive and negative control drugs to evaluate the performance of our methods. Our methods have AUCs that are often substantially higher than the AUCs of a baseline method that only uses demographic characteristics for confounding control. Our methods also give confidence intervals for causal effect parameters that cover the expected no effect value substantially more often than this baseline method. The case-control study design, unlike the self-controlled study design, can be used in the fairly typical setting of EHR databases without longitudinal information on patients. With our variable selection method, these databases can be more effectively used for the detection of ADRs.

## Introduction

To enable doctors, patients and regulators to make informed decisions regarding the costs and benefits of drugs, it is important to thoroughly understand the risk that they pose of adverse drug reactions (ADRs). Randomized clinical trials provide much of the available information on the safety of a drug in humans before regulatory bodies approve the drug. However, these studies are often underpowered to detect rare ADRs, ADRs occurring in patients with comorbidities or taking other medications that may have been underrepresented in the clinical trials, or ADRs that occur after drug use of long duration. Therefore, post-marketing surveillance of drugs is required to supplement the information learned prior to approval in clinical trials [1].

Continuing surveillance for ADRs has commonly been conducted using spontaneous reporting systems, which contain reports of suspected ADRs by medical professionals and consumers. These systems have limitations, including underreporting and biased reporting [2, 3]. An alternative tool for this surveillance that has been the subject of recent research, and which facilitates a complementary method for detection of ADRs, is the use of administrative claims and electronic health record (EHR) data in observational studies to retrospectively identify drugs that cause ADRs (for a review, see [4], chapter 14).

One threat to the validity of observational studies of drug safety is the difficulty in determining if an observed association between the use of a drug and the occurrence of a health outcome of interest (HOI) is due to a causal relationship between the drug and the HOI or to confounding by some difference between the groups of patients being compared. In a cohort study design, for example, the occurrence of an HOI might be compared between two groups of patients: one exposed to drug A and the other to drug B. If the patients taking drug A are sicker on the whole than the patients taking drug B, any higher rate of the HOI in the group taking drug A might be due to this group being sicker, rather than to an adverse effect of drug A.

The principal strategy for reducing the effect of confounding in observational studies is to control via matching or regression for various characteristics of patients in the two groups, such as age, sex and health status, in order to assure that the groups being compared are similar [5–7]. Although this strategy can be effective in reducing confounding, it may not be fully effective if some important characteristic varying between the two groups, such as smoking status, cannot be controlled for, perhaps because it was never measured.

The Observational Medical Outcomes Partnership (OMOP) carried out an extensive study to compare the efficacy of various different observational study designs and analytic methods in combating the harmful effects of confounding [8]. To provide a means of testing these methods, OMOP established a set of known drug-outcome causal relationships, including both positive controls, drugs where evidence exists to suspect a positive causal relationship with a health outcome of interest (HOI), and negative controls, drugs where no such evidence exists [9]. OMOP then used this set to test the performance of several study designs and analytic methods to detect ADRs from administrative claims and EHR data (for a discussion of the different kinds of claims and EHR data used in observational studies of drug safety, see [10]). One finding of OMOP's study was that self-controlled study designs were the most effective among the designs tested [1, 8]. These study designs compare the incidence of a HOI during a period of time in which a subject is exposed to a drug to the incidence of the HOI during a different period of time in which the same subject is not exposed to the drug. Therefore, in this study design, each individual serves as his or her own control. Since individuals have many characteristics, such as comorbidities, that often do not change across time, the control of confounding in this design can be highly effective. In the case-control study design, by contrast, persons with an HOI are compared to an independent control group without the HOI. OMOP found the case-control study design to be less effective at reducing confounding than the self-controlled study design, likely due to the fact that this independent control group can vary from the cases quite substantially. In fact, the case-control designs in OMOP's study performed quite poorly, often no better than chance in terms of distinguishing positive from negative controls [11]. OMOP's implementation of the case-control design, however, controlled only for a few demographic characteristics, and not for other characteristics of the subjects.

Due to the fragmented nature of health care delivery, many available databases, such as the EHR at NewYork-Presbyterian/Columbia University Medical Center (NYP), only contain a small part of a patient's health record, limited in time and also in scope. For example, some patients may visit practitioners at several different health systems, and their records may be divided among more than one EHR system. An advantage of the case-control study design is that it can be used with such fragmentary EHR data to detect ADRs. By contrast, the self-controlled study design can only be used where longitudinal patient data, *i*.*e*., data documenting patient conditions and drug exposures over an extended period of time, is available, as might be the case for data from a health maintenance organization. Our goal in this paper is to revisit the use of the case-control study design, to study whether improved analytic methods that use patient information beyond demographic characteristics could effectively be used with such fragmentary EHR data to detect ADRs.

As with the use of EHRs in observational studies generally, there are numerous open research issues regarding the use of the case-control study design to detect ADRs from EHR data. For example, there are challenges in the selection of cases and matching controls. Controls often tend to have less serious conditions than cases and therefore they have different distributions of confounding factors. Also, because controls have less serious conditions than cases, they have fewer interactions with EHR systems, and so the completeness of available data regarding their healthcare history will vary systematically from that of cases. Generally, better results will be obtained if case and control populations are restricted so that cases and controls are more similar with respect to confounders [7]. A countervailing factor, however, is that the power to detect ADRs, all other things being equal, is lower with smaller case and control populations. Here we only consider the inpatient population, to avoid confounding that would arise if a largely inpatient case population were compared with a largely outpatient control population.

There are also challenges in the selection of covariates to control for in the analysis of case-control study data. Some studies, like that of OMOP, only control for demographic characteristics. This entails a significant loss of information, but selecting additional covariates to control for is challenging since there are thousands of potential covariates to choose from. There is a significant body of research considering variable selection with high-dimensional data [12, 13]. The OMOP reference set provides a unique opportunity to measure the efficacy of various variable selection strategies with real data rather than with simulated data, which often lack the complex biases present in real data.

In previous work, we have experimented with the LASSO, a popular variable selection strategy, to select appropriate confounders for adjustment in case-control studies. In our first study of ADRs, we applied a one-step LASSO variable selection strategy, using as confounders comorbid conditions positively associated with the HOI and the drug of interest [14]. In a subsequent study, in the context of the development of a method to combine signals for ADR detection from observational healthcare data and from spontaneous reporting systems, we modified our previous method by using as confounders comorbid conditions associated, positively or negatively, with the HOI and the drug of interest [15]. We also implemented a novel two-step LASSO variable selection strategy, motivated by theoretical considerations, to reduce the false positive rate. In the principal contribution of this paper, we show, using the OMOP reference set, that these LASSO methods can effectively use rich data on comorbidities to significantly outperform methods, like that tested by OMOP, that only control for demographic variables. We conclude that a case-control method that properly selects comorbidities to control for shows promise for the detection of ADRs from EHR data, even if it the data is fragmentary and incomplete.

## Methods

### Data

This study was conducted using a database of EHR data from NYP, with approval from the Columbia University Medical Center Institutional Review Board (protocol number AAAD6669). EHR data used consisted of i) structured medication data obtained using the MedLEE natural language processing system from admission and discharge notes for inpatient visits [16], ii) coded demographic information and iii) diagnosis codes from the International Statistical Classification of Diseases and Related Health Problems, Version 9 (ICD-9 codes), all from the period January 1, 2004 through December 31, 2012.

Complete EHR data for all inpatient and outpatient visits from NYP for this period is not available in our database, and for some visits, some kinds of data are available while others are not. For example, for some visits we have ICD-9 codes but no notes, and therefore lack medication data. We use inpatient notes from the NYP EHR starting from January 1, 2004, but the percentage of inpatient visits with ICD-9 codes that have an associated note is lower in earlier years than in later years.

NYP is a tertiary health center, and for many patients, we only have information from one or two visits to NYP. In total, there are 294,271 patients with one or more inpatient or outpatient notes in our database during the study period; 126,711 of these patients (43.1%) have only one inpatient visit (and no outpatient visits) with a note. Over the study period, there are 376,624 inpatient visits with notes in our database, and 963,785 outpatient visits with notes. Although we have more outpatient visits with notes, we have more information from each inpatient visit. For example, the average number of ICD-9 codes per inpatient visit is 8.45; per outpatient visit the average number is 2.32.

### Method overview

**Fig 1:**
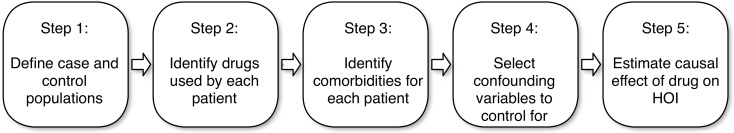
Illustration of the five steps of our analysis workflow.

We examine four HOIs in this study: acute kidney injury (AKI), acute liver injury (ALI), acute myocardial infarction (AMI) and gastrointestinal ulcer hospitalization (GIU). We choose these HOIs since they are the HOIs for which OMOP has established its set of known drug-HOI causal relationships, which we use to measure the performance of our methods [9]. To measure the causal effect of a drug on a HOI, we 1) define case and control populations, 2) identify the drugs used by each patient, 3) identify comorbidities and demographic characteristics for each patient, 4) select confounding variables to control for and 5) use logistic regression to estimate the causal effect of the drug on the HOI. We measure this causal effect with the ratio of the odds of having the HOI between those patients taking the drug and those patients not taking the drug, with control for the selected confounding variables. We will now describe each of these steps in detail. Our analysis workflow is summarized in Fig 1.

#### Step 1. Defining case and control populations

For each HOI, we use OMOP's broad definition of the HOI, which consists of a list of ICD-9 codes, to define case and control populations [17]. The case population for each HOI consists of patients with at least one ICD-9 code matching this list. The control population consists of all of the other patients. For some HOIs, OMOP's broad definition requires that some ICD-9 codes—*e*.*g*., all the codes for GIU—coincide with an inpatient hospitalization or ER visit. Our data is more complete for inpatient hospitalizations than for outpatient visits, many of which lack a clinical note in our database. In addition, there are systematic differences in medication use and number of comorbid conditions recorded in our database for inpatient hospitalizations and outpatient visits. Since our cases have serious conditions and so are more likely to be inpatients, to obtain an appropriate control population we only include controls with an inpatient visit with a note. We only include cases if their first ICD-9 code that matches OMOP's list coincides with an inpatient admission for which we have a note, so that we can discover, from the section of the note that describes medications taken prior to admission, which medications they were taking immediately before they developed the HOI. For this reason, the sum of the number of cases and controls varies across the 4 HOIs.

Table 1 compares various statistics for the case and control populations for each of the 4 HOIs constructed as described above. The case populations are older on average than the control populations, with a higher number of inpatient visits, medications and ICD-9 codes. Two patient populations highly over-represented among the controls, as compared to the cases, are newborns and pregnant women. The case populations for ALI and GIU are much smaller than for AKI and AMI; combined, the combined ALI and GIU case populations are only approximately 30% as large as the combined AKI and AMI case populations.

**Table 1:** Characteristics of case and control populations for 4 HOIs.

	AKI		ALI		AMI		GIU	
	Control	Case	Control	Case	Control	Case	Control	Case
# patients	198,458	22,785	211,365	6,832	200,154	18,389	214,289	4,603
Mean # inpatient visits	1.1	1.37	1.13	1.38	1.13	1.16	1.13	1.47
Mean # outpatient visits	0.19	0.18	0.19	0.16	0.19	0.11	0.18	0.16
Median # medications	16	50	18	39	17	32	18	39
Median # ICD-9 codes	20	55	21	46	20	32	21	50
Median age	44.69	68.42	47.92	56.37	44.17	67.34	47.95	63.24
% pregnant	7.3	0.2	6.7	2.0	7.3	0.0	6.7	0.3
% age less than 1	18.1	0.8	16.6	5.5	18.0	0.1	16.6	2.2

Statistics are for the 180 days ending on the index admission, so the mean number of inpatient visits includes the index admission. Ages are as of the index admission.

#### Step 2. Identify drugs used by each patient

After we identify case and control populations, we identify the drugs used by each case and control from admission and discharge notes. We use the MedLEE natural language processing system to identify drug names (*e*.*g*., Lipitor) in narrative text in notes and RxNorm to normalize them to their generic names (in the case of Lipitor, atorvastatin) [18]. MedLEE also identifies modifiers, such as time and negation, so drugs mentioned in the note but that were discontinued, not taken by the patient, or that were taken in the past by the patient are excluded. In addition, drugs recorded in the note due to patient allergies are identified and excluded.

For cases, we want to extract drugs taken currently by patients before the onset of the HOI, so that we can detect drugs causing the HOI. To do this, we use only drugs extracted from admission or discharge notes associated with the first admission of the patient with an ICD-9 code matching OMOP's definition of the HOI. Since we have more information for later admissions than for earlier admissions, it is preferable that the dates of admissions on which medication usage information is collected for cases and controls be similarly distributed, to avoid systematic differences in apparent medication use driven by differences in data completeness for cases and controls. For each control, we therefore use drugs from admission and discharge notes associated with a randomly selected inpatient admission for that control with an admission or discharge note. We call the inpatient admission with respect to which we collect medication usage information for each case or control the “index admission.” Our method of randomly selecting the index admission for each control patient results in a distribution of index admission dates that is roughly similar for cases and controls. However, more index admissions for controls are in later years than for cases, since for cases the index admission is the first inpatient admission with a matching ICD-9 code for the HOI, and there may be several such admissions for a patient.

MedLEE tags drugs extracted from notes with the name of the section in the notes from which they are extracted. In order to exclude drugs potentially prescribed in response to the HOI, we only include drugs associated with the index admission if they are in the “home medications” or the “medications on admission” sections of the notes. We therefore exclude sections like “assessment,” “history of present illness,” and “hospital course” which may be more likely to include drugs related to treatment of the HOI. We also exclude the “medications” section since it is ambiguous and, especially for a discharge note, this section might include medications prescribed during the hospital stay. In order to avoid systematic differences in apparent medication use driven by differences in data completeness for cases and controls, we apply the same criteria for inclusion of medications to both cases and controls. To test the robustness of our analytical methods to differences in the ways our case-control datasets are constructed, we also generate a second dataset using different sections of the notes, and evaluate our methods on that dataset. This second dataset is discussed in the supplement.

#### Step 3. Identify comorbidities and demographic characteristics

Although appropriate covariates to adjust for could be elicited from the medical literature for every drug-HOI pair, our goal is to test the potential of methods that automatically detect appropriate covariates from the EHR data. Such methods could provide an efficient and easily replicable way to test drug-HOI pairs for a causal relationship and adjust for important covariates that might not be mentioned in the literature. Therefore, we collect a rich set of covariates for each patient, and we then select among these covariates in the following step of the analysis.

We use demographic variables, including race, sex, age at the index admission (treated, after binning, as a categorical variable), and year of the index admission (treated as a categorical variable). For each patient, we also use ICD-9 codes associated with the index admission for each patient and with prior visits, excluding those that match OMOP's definition of the HOI. We recode the ICD-9 codes using PHEWAS, which is a grouping of ICD-9 codes that aggregates similar clinical conditions [19]. Whereas there are more than 15,000 unique ICD-9 codes in our dataset, there are fewer than 1,700 unique PHEWAS codes. As an example of this recoding, PHEWAS groups twenty different ICD-9 codes, including 200.6 (Anaplastic large cell lymphoma) and 200.7 (Large cell lymphoma), together into a single “Large cell lymphoma” category. This reduction in the number of codes makes the succeeding steps of our method less computationally demanding.

More than 15% of the ICD-9 (non-unique) codes in our database have no PHEWAS equivalent, and so the information in these codes is lost when using PHEWAS. Most of the ICD-9 codes with no PHEWAS equivalent are E- (external causes of injury) and V- (supplemental classification) codes, referring, for example, to pregnancy status or to a routine pediatric visit. To avoid the loss of some of this information when using PHEWAS codes, we create groups of i) normal pregnancy, ii) high-risk pregnancy, iii) child health supervision, iv) contraceptive management and v) HIV counseling ICD-9 codes, and include a composite code for each such group along with the PHEWAS codes. After this grouping, only about 10% of the non-unique ICD-9 codes still have no PHEWAS or composite code equivalent.

#### Step 4. Select confounding variables to control for

We examine several different methods for covariate selection. Here we describe two of these methods, which we call the “1-step LASSO” methods and “1 model per HOI” methods. In the supplement we describe several variations of these methods. The “1-step LASSO” method may be summarized as follows:

- Step 4A: Rank available covariates based on the significance of their correlation with case/control status or drug use and select covariates whose correlation is significant at the 5% level.
- Step 4B: Use LASSO logistic regression of case-control status against covariates selected in step 4A and drug use to select covariates.

In the first step (Step 4A), we rank all the covariates based on their correlation with both case/control status and drug use, and select the covariates whose correlation with either case/control status or drug use is significant at the 5% level. As a running example, consider measuring the causal effect of the negative control drug simethicone, used to control bloating, on AKI. The marginal odds ratio between AKI and simethicone, using medications only from the index admission date, is 3.19 (p-value<0.0001). There are 1,694 covariates in the case-control data set before variable selection. The covariate most significantly correlated with AKI is chronic renal failure, and the covariate most significantly correlated with simethicone is flatulence. In total, there are 1,299 covariates whose correlation with either AKI or simethicone use is significant at the 5% level (543 are significantly correlated with simethicone, 1,235 with AKI). These covariates are carried forward to the second step.

In the second step (Step 4B), we use LASSO logistic regression of case-control status against the covariates selected in step 1, as well as the drug of interest, to select covariates that predict case/control status well. LASSO logistic regression carries out logistic regression under the constraint that the sum of the absolute values of the regression coefficients be less than the value of a given threshold. This forces the values of some of the coefficients to zero. The lower the threshold, the more coefficients are forced to zero. The covariates with non-zero coefficients are the ones selected by the LASSO. We use 5-fold cross validation to select the optimal value of the threshold. In 5-fold cross validation, at each level of the threshold that is considered, the dataset is divided into 5 pieces. Each of these pieces in turn serves as a test dataset. Using only the other 4 pieces of the dataset, a LASSO model is fit and used to predict the case-control status of all the patients in the test dataset (which is not used in model fitting). The deviance, a statistical measure of how well the model fits the observed case-control status of the patients, is then calculated with respect to the test dataset. The optimal threshold is that threshold at which the average deviance on the 5 test datasets is lowest.

In the regression of AKI status on the 1,299 covariates selected as described above, 684 (including chronic renal failure, mentioned above) have non-zero coefficients at the optimal threshold value. It is important to note that in this step, the OMOP reference set is not used; what is being optimized is the ability of the LASSO model to predict the case-control status of the patients using their demographic characteristics and comorbidities, not the ability of the model to correctly predict if a drug causes an HOI or not. The OMOP reference set labels (*i*.*e*., positive or negative control) is only used during evaluation, not during Steps 1 through 5 of our method.

#### Step 5. Use logistic regression to estimate causal effect of drug on HOI

Finally, using as explanatory variables the variables selected as above, as well as the drug of interest, we use logistic regression, using case/control status as the response variable, to estimate the ratio of the odds of having the HOI between those patients taking the drug and those patients not taking the drug, along with its associated confidence interval. In the regression of AKI against the covariates selected above as well as simethicone, we estimate the conditional odds ratio of having AKI between patients taking and not taking simethicone as 1.08, and the two-sided 95% confidence interval as [0.71–1.61]. The value 1 (indicating equal odds of having AKI between patients taking and not taking simethicone) is well within this confidence interval, so we have little evidence that simethicone causes AKI.

### “1 model per HOI” method

In the “1 model per HOI” method, in lieu of fitting a separate logistic regression model for each drug-HOI pair, we fit a single logistic regression model in Step 5 that includes all drugs being tested. In this model, the estimates for each drug will be adjusted for use of the other drugs. In this method, we also modify Steps 4A and 4B by only using case-control status in the selection of covariates, not drug use.

### Baseline models used for comparison

We compare our variable selection methods to two different baseline models. In the “Only demographic” method, similar to that used in OMOP's study, and which we use as a proxy for comparing our analytical methods to those used by OMOP, we adjust only for demographic covariates, not comorbidities [11]. In the other baseline method, which we call “No adjustment”, we do not adjust for any covariates. For this model, we do not need to fit a logistic regression model. Instead, we use the odds ratio between use of the drug and case/control status (the marginal odds ratio) to estimate the causal effect of the drug on the HOI.

All the analytical methods we examine here are summarized in Table 2. In the supplement we discuss other methods, including (i) a method that uses a more complex 2-step LASSO variable selection, (ii) a method that uses ICD-9 codes instead of PHEWAS codes, (iii) a method that omits the variable pre-screening step, Step 4A, (iv) a cross-validation method that selects a different LASSO model less complex than the one selected using the optimal threshold and (v) a method that excludes cases and controls to create better matched case and control populations prior to Steps 4 and 5.

**Table 2:** Analytical methods examined.

	Covariates used	Covariate selection method	Estimation method
No adjustment	None	None	Marginal odds ratio
Only demographic	Demographic	1-step LASSO	1 model per drug-HOI pair
1-step LASSO	PHEWAS and demographic	1-step LASSO	1 model per drug-HOI pair
1 model per HOI	PHEWAS and demographic	1-step LASSO	1 model per HOI

### Evaluation design

To evaluate the performance of each of these methods, for each HOI we run each method on OMOP's reference set of positive controls, drugs suspected to have a causal effect on the HOI, and negative controls, drugs for which there is no evidence of a causal effect on the HOI [9]. There are a total of 165 positive control-HOI pairs and 234 negative controls-HOI pairs in this reference set. Many of these drugs, however, were used by very few of the patients in our database. We therefore restrict our attention to those drugs for which we have 50% power to detect a marginal unadjusted odds ratio of 1.9 (close to the upper range of the detected odds ratios in our experiments below) in cases versus controls, with a Type I error rate of 2.5%. This leaves 52 positive control-HOI pairs and 42 negative control-HOI pairs.

For each drug-HOI pair, we use either the logistic regression results or Fisher's exact test, for marginal odds ratios, to calculate the probability, under the null hypothesis of an odds ratio of 1 between drug use and case/control status, that we would observe a causal effect as positive or more positive than that actually observed. We use these one-sided p-values as a measure of the drug's causal effect on the HOI, and use a threshold of 0.025 (corresponding to a two-sided p-value of 0.05) to define drug-HOI causal relationships detected by our methods.

To evaluate and compare the performance of these methods, we use the one-sided p-values generated by each method as a binary classifier and measure the area under the curve (AUC), a measure of predictive accuracy. The AUC is equal to the probability that a classifier will rank a randomly chosen positive control higher than a randomly chosen negative control. A method that perfectly distinguishes positive controls from negative controls (regardless of the threshold that divides the positive and negative controls) would yield an AUC of 1; random guessing would yield on average an AUC of 0.5. In practice, when using a classifier, one might pick a single threshold to distinguish positive instances from negative instances. The AUC allows an evaluation that does not depend on a specific choice of threshold; it is instead a composite measure that averages over all thresholds.

For the negative controls, which we expect to have no effect on the HOI, we also measure how often two-sided 95% confidence intervals for the odds ratio of the HOI, comparing subjects taking and not taking the drug, include the expected no effect value of 1 [1]. We refer to this metric as the “coverage probability under the null”. If a method accurately estimates the causal effect of drugs on HOIs, these confidence intervals should include the expected no effect value approximately 95% of the time. For example, using the “1-step LASSO” method, the estimated confidence interval for the odds ratio of AKI and the negative control simethicone includes the expected no effect value of 1 for the odds ratio.

## Results

Table 3 shows the “No adjustment”, “Only demographic”, “1-step LASSO” and “1 model per HOI” results for all four HOIs.

**Table 3:** Effect of adjusting for demographic characteristics and comorbidities.

HOI	Experiment type	AUC	Positive controls with one-sided p-value < 0.025	Negative controls with one-sided p-value < 0.025	Negative controls with 95% CI including null
**AKI**	No adjustment	0.65	12/13 (92%)	10/12 (83%)	2/12 (17%)
	Only demographic	0.54	11/13 (85%)	9/12 (75%)	3/12 (25%)
	1-step LASSO	0.88	8/13 (62%)	1/12 (8%)	11/12 (92%)
	1 model per HOI	0.83	6/13 (46%)	1/12 (8%)	11/12 (92%)
**ALI**	No adjustment	0.38	11/20 (55%)	3/5 (60%)	2/5 (40%)
	Only demographic	0.40	9/20 (45%)	3/5 (60%)	2/5 (40%)
	1-step LASSO	0.40	2/20 (10%)	1/5 (20%)	4/5 (80%)
	1 model per HOI	0.51	2/20 (10%)	1/5 (20%)	4/5 (80%)
**AMI**	No adjustment	0.77	8/10 (80%)	6/17 (35%)	9/17 (53%)
	Only demographic	0.88	6/10 (60%)	1/17 (6%)	15/17 (88%)
	1-step LASSO	0.95	3/10 (30%)	0/17 (0%)	17/17 (100%)
	1 model per HOI	0.93	3/10 (30%)	0/17 (0%)	17/17 (100%)
**GIU**	No adjustment	0.50	8/9 (89%)	8/8 (100%)	0/8 (0%)
	Only demographic	0.40	8/9 (89%)	7/8 (88%)	1/8 (12%)
	1-step LASSO	0.57	4/9 (44%)	3/8 (38%)	5/8 (62%)
	1 model per HOI	0.65	2/9 (22%)	2/8 (25%)	6/8 (75%)

The final column of this table indicates for how many negative control drugs the two-sided 95% confidence interval for the odds ratio of the effect of the drug on the HOI includes the expected no effect value of 1. A negative control drug that has a one-sided p-value > 0.975 will not be counted in the numerators of either of the final two columns of this table.

### Performance without adjustment

Without adjustment, AUCs vary widely among the four HOIs, so that a classifier based on the “No adjustment” method performs better than chance for AMI and AKI, about as well as chance for GIU, and worse than chance for ALI. In OMOP's analyses of observational methods for detection of ADRs, in general AUCs were found to be lowest for ALI and highest for AKI, with AMI and GIU in between [1]. Here too, as in OMOP's results, AUCs are lowest for ALI, and also low for GIU. Both of these are HOIs for which we have many fewer cases than for AKI and AMI.

Without adjustment, coverage probabilities under the null are quite poor. For AKI, for example, the lower bounds of 95% confidence intervals for the odds ratio for 10 of the 12 negative controls (83%) are greater than 1, so that they are all estimated to cause AKI.

### Performance with only demographic variables

The “Only demographic” method is comparable to the case-control analytic method which was found by OMOP to perform poorly [11]. In terms of AUC, the “Only demographic” method performs better than the “No adjustment” method for AMI, and about as well or worse for the other three HOIs. In terms of coverage probabilities under the null, for all HOIs other than AMI, coverage probabilities under the null using this method are substantially below the nominal level of 95%, and are often the same or only marginally better than without adjustment.

### Performance with “1-step LASSO”and “1 model per HOI”methods

**Fig 2:**
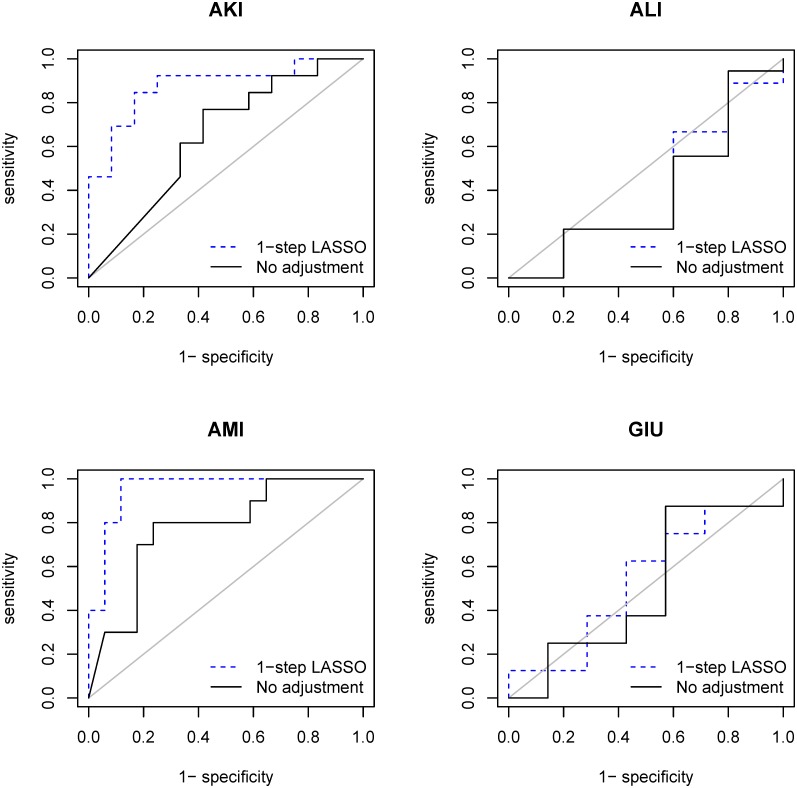
Receiver operating characteristic curves for the “1-day LASSO” method and the “No adjustment” method.

With the “1-step LASSO” method coverage probabilities under the null are consistently improved relative to the “No adjustment” and “Only demographic” methods, for AKI and AMI coming very close to the nominal levels of 95%. AUC values are substantially increased relative to the “No adjustment” and “Only demographic” methods for AKI. For AMI, the AUC is increased relative to the two baseline methods, and the negative controls are nearly perfectly distinguished from the positive controls; however, only three of the ten positive controls (nifedipine, rosiglitazone and indomethacin) have a one-sided p value below a threshold of 0.025, corresponding to the commonly used two-sided p-value threshold of 0.05. For GIU and ALI, unlike the coverage probabilities under the null, AUC values are generally not substantially improved relative to the baseline methods. Receiver operating characteristic curves showing sensitivity versus specificity for the “1-step LASSO” method as compared to the “No adjustment” method are in Fig 2.

Results are generally similar for the “1-step LASSO” and “1 model per HOI” methods. For GIU and ALI, however, AUC values are higher for the “1 model per HOI” method.

### False positive results

Estimates and confidence intervals for odds ratios for all false positive results for the “1-step LASSO” and “1 model per HOI” methods, are shown in Table 4. Except for fluticasone-GIU, the drug-HOI pairs that are false positives for the “1-step LASSO” method are also false positives for the “1 model per HOI method.”

**Table 4:** False positive results for “1-step LASSO” and “1 model per HOI” methods.

HOI	drug	1-step LASSO Estimate (Confidence interval)	1 model per HOI Estimate (Confidence interval)
AKI	darbepoetin alfa	1.77 (1.16–2.69)	1.57 (1.03–2.4)
ALI	lactulose	2.91 (2.19–3.87)	2.66 (2.00–3.54)
GI	fluticasone	1.34 (1.04–1.72)	0.67 (0.38–1.16)
GI	rosiglitazone	2.10 (1.32–3.36)	1.96 (1.22–3.14)
GI	salmeterol	1.60 (1.22–2.11)	2.03 (1.11–3.70)

## Discussion

Our results demonstrate that the case-control study design, when used with the variable selection methods examined here, can be effective for estimating causal effects of drugs on HOIs. The number of positive and negative controls examined is low due to the low number of patients in the NYP EHR taking many of the drugs in the OMOP reference set. However, for the drugs examined coverage probabilities under the null are close to the nominal values, unlike the coverage probabilities for many of the analytic methods and study designs examined by OMOP [1]. In addition, the AUC values for AKI and AMI are quite favorable. For many positive controls the confidence intervals for our positive estimates are relatively wide, indicating uncertainty about these estimates, and whether or not they are positive. For example, for AMI, the AUC value for the “1-step LASSO” method is very high, but only three of the positive controls have a one-sided p value below a threshold of 0.025, corresponding to the commonly used two-sided p-value threshold of 0.05 and suggesting inadequate power to statistically identify an actual causal effect using our dataset.

As opposed to the “1-step LASSO” and “1 model per HOI” methods, the “Only demographic” method that adjusts only for demographic information and is similar to the analytic method used by OMOP in its case-control studies had poor performance, consistent with the poor performance described by OMOP [11]. The contrast in performance with our variable selection methods illustrates the benefit of using the rich information about patients available in the EHR to adjust case-control estimates of causal effects for confounding, and raises the possibility that case-control studies can be an effective complement to the self-controlled case study, especially for fragmentary and incomplete sources of healthcare data.

Many of the covariates automatically selected by our variable selection methods are clinically reasonable. For example, when only demographic characteristics are adjusted for, then lipase is estimated to cause AKI, even though lipase-AKI is an OMOP negative control. The confidence interval for the odds ratio of lipase on AKI with the “Only demographic” method is [2.10, 6.06]. After adjustment, however, the confidence interval is [0.67, 9.81]. One of the variables selected for in the “1 model per HOI” model for AKI is acute pancreatitis, which is reasonable in estimating the lipase-AKI odds ratio, since a lipase test is ordered when acute pancreatitis is suspected, and AKI is a common complication of acute pancreatitis [20].

As another example, when only demographic characteristics are adjusted for lactulose is estimated to cause AKI and GIU. The confidence interval for the odds ratio of lactulose on AKI is [3.99, 5.63] and on GIU is [3.68, 6.22]. After adjustment, the corresponding confidence intervals are [0.86, 1.37] and [0.69, 1.39]. Two of the variables selected for in the “1 model per HOI” model for both AKI and GIU are Chronic liver disease and cirrhosis and Chronic nonalcoholic liver disease. Lactulose is a common treatment for hepatic encephalopathy, the loss of brain function that occurs when the liver doesn't remove toxins from the blood. Hepatic encephalopathy is a syndrome that often accompanies chronic liver disease, which in turn is known to be associated with both AKI and GIU [21, 22]. Chronic liver disease and Chronic nonalcoholic liver disease are therefore clinically reasonable variables to control for in estimating the lactulose-AKI and lactulose-GIU odds ratios.

As a final example, when only demographic characteristics are adjusted for, sitagliptin is estimated to cause AMI, with a confidence interval for the odds ratio of [1.21, 1.77]. Sitagliptin is used in the treatment of diabetes, and diabetes patients are known to be at high risk for AMI [23]. Diabetes is one of the variables selected for in the “1 model per HOI” model for AMI; after adjustment the sitagliptin-AMI odds ratio is [0.77, 1.21].

A significant benefit of our method, shared by self-controlled study designs, is that it is largely automatic, not requiring expert selection of covariates to use for adjustment. When assembling a data set for analysis using any method, however, it is important to consider potential biases induced by design choices, like the choice of which patients to include as cases and controls, and which medications to use, both in terms of selecting the index admission date and in terms of selecting which medications from the clinical notes to include [7]. Here these potential biases influenced many of our study design choices, like our decision to only include inpatients as cases and controls, our stringent limits on the sections of clinical notes from which we used medications, and our application of the same data inclusion criteria to both cases and controls.

Performance of our methods is poorer for ALI and GIU than it is for AKI and AMI. Apart from the smaller sample sizes in our dataset for ALI and GIU, this may be due to our use of ICD-9 codes to distinguish between cases and controls. ICD-9 codes may be highly unreliable, and their reliability has been shown to depend significantly on the condition being coded. For example, a study comparing free text in notes with ICD-9 codes showed high concordance for AMI, relatively low concordance for AKI and very poor concordance for pneumonia [24]. Given the unreliability of ICD-9 codes, an unknown number of the ALI and GIU controls may have actually had ALI and GIU, thereby limiting our power to detect drug-HOI associations.

The promising performance of the “1 model per HOI” model for AKI and GIU, although not substantially different from the performance of the other methods, suggests the potential benefit of selecting additional medications to use for adjustment. For example, as illustrated in Table 4, after adjusting for other drugs, fluticasone no longer has an estimated causal effect on GIU. This is likely due to the adjustment for salmeterol use, since fluticasone and salmeterol are often co-administered in an inhaler. Similar to the recoding of ICD-9 codes using PHEWAS codes used here, medications could be grouped using their mechanism of action or indication to reduce the dimensionality of the covariate data, but that would require additional knowledge engineering.

Another avenue that could be explored in future work is exploration of more complex models that loosen the linear assumption of the logistic regression model. For example, covariates indicating the co-occurence of two comorbidities (so-called “interaction terms”) could be added to the logistic regression model, to allow models to accommodate non-additive effects of comorbidities. As seen above, however, some ICD-9 and PHEWAS codes already indicate the co-occurrence of comorbidities (for example, “Anemia in chronic kidney disease”). There may therefore not be much to be gained from including other interaction terms. Including interaction terms would exponentially increase the number of potential covariates, and so would be computationally demanding.

Our study is limited by the small sample size in NYP EHR, and the small numbers of patients taking some drugs, which also limited the numbers of drugs from the OMOP reference set that we could use for evaluation. Further work could potentially increase the sample size and the precision of our estimates by also using outpatient data, although care would be required to avoid bias resulting from different data quality for inpatient and outpatient visits.

As we have demonstrated, using solely demographic information for adjustment yields, as in OMOP’s study, results with poor statistical properties. Using automatic variable selection to select comorbidities to use as covariates provides much better results, and makes the case-control method a viable method for identifying drugs that may cause ADRs.

## Conclusion

We have shown the promise of using data on comorbidities together with automatic variable selection strategies to confront the problem of confounding in ADR detection using EHR data and the case-control study design. Using OMOP's reference set of established drug-HOI causal relationships, we have shown the potential for substantial benefits from using comorbidities for confounding control, as opposed to just using demographic data as in OMOP's examination of the case-control method. Unlike the self-controlled study designs that OMOP's study found to be most effective, the case-control study design, in conjunction with our methods, can be used in the setting of databases with fragmentary healthcare information. Our study provides a roadmap for effectively using these databases for the detection of ADRs.

## Supporting Information

S1 File Discussion of other analytic methods and other dataset. (DOCX)

S2 File Results for all analytical methods. (TXT)
