# Supplementary material for: Using Rich Data on Comorbidities in Case-Control Study Design with Electronic Health Record Data Improves Control of Confounding in the Detection of Adverse Drug Reactions
Source: PLoS One. 2016 Oct 7;11(10):e0164304. doi: 10.1371/journal.pone.0164304 (PMC5055309; doi:10.1371/journal.pone.0164304)
Supplement: S1 File — (DOCX) [file pone.0164304.s001.docx]

**S1 File. Supporting information for “Using Rich Data on Comorbidities in Case-Control Study Design with Electronic Health Record Data Improves Control of Confounding in the Detection of Adverse Drug Reactions”**

**S1 Table. Case and control populations.** There are 223,795 patients in the study period with at least one inpatient visit. The sum of the numbers in the second and fourth columns in the table below in each row is 223,795.

| **HOI** | **Number of patients with a matching ICD-9 code and at least one inpatient visit (cases)** | **Number of patients with a matching ICD-9 code whose first matching ICD-9 code coincides with an inpatient visit** | **Number of patients with no matching ICD-9 codes and at least one inpatient visit (controls)** |
| --- | --- | --- | --- |
| **AKI** | 25,337 | 22,785 | 198,458 |
| **ALI** | 12,430 | 6,832 | 211,365 |
| **AMI** | 23,641 | 18,389 | 200,154 |
| **GI** | 9,506 | 4,603 | 214,289 |

**Other analytical methods examined**

The other analytical methods we examine are the “2-step LASSO” method, the “ICD-9” method, the “1-se” method, the “No screening” method and the “Pre-matching” method. Results for these methods are presented in S2 Table and S2 Fig.

**S2 Table. Results for additional analytic methods.**

| **HOI** | **Experiment type** | **AUC** | **Positive controls with one-sided p-value < 0.025** | **Negative controls with one-sided p-value < 0.025** | **Negative controls with 95% CI including null** |
| --- | --- | --- | --- | --- | --- |
| **AKI** | 1-step LASSO | 0.88 | 8/13 (62%) | 1/12 (8%) | 11/12 (92%) |
|  | 2-step LASSO | 0.88 | 8/13 (62%) | 1/12 (8%) | 11/12 (92%) |
|  | 1 model per HOI | 0.83 | 6/13 (46%) | 1/12 (8%) | 11/12 (92%) |
|  | ICD-9 | 0.79 | 5/13 (38%) | 0/12 (0%) | 12/12 (100%) |
|  | 1-se | 0.83 | 6/13 (46%) | 1/12 (8%) | 11/12 (92%) |
|  | No screening | 0.81 | 6/13 (46%) | 1/12 (8%) | 11/12 (92%) |
| **ALI** | 1-step LASSO | 0.4 | 2/20 (10%) | 1/5 (20%) | 4/5 (80%) |
|  | 2-step LASSO | 0.39 | 2/20 (10%) | 1/5 (20%) | 4/5 (80%) |
|  | 1 model per HOI | 0.51 | 2/20 (10%) | 1/5 (20%) | 4/5 (80%) |
|  | ICD-9 | 0.5 | 2/20 (10%) | 1/5 (20%) | 4/5 (80%) |
|  | 1-se | 0.55 | 1/20 (5%) | 1/5 (20%) | 4/5 (80%) |
|  | No screening | 0.53 | 2/20 (10%) | 1/5 (20%) | 4/5 (80%) |
| **AMI** | 1-step LASSO | 0.95 | 3/10 (30%) | 0/17 (0%) | 17/17 (100%) |
|  | 2-step LASSO | 0.95 | 3/10 (30%) | 0/17 (0%) | 17/17 (100%) |
|  | 1 model per HOI | 0.93 | 3/10 (30%) | 0/17 (0%) | 17/17 (100%) |
|  | ICD-9 | 0.88 | 2/10 (20%) | 0/17 (0%) | 17/17 (100%) |
|  | 1-se | 0.93 | 3/10 (30%) | 0/17 (0%) | 17/17 (100%) |
|  | No screening | 0.94 | 3/10 (30%) | 0/17 (0%) | 17/17 (100%) |
| **GIU** | 1-step LASSO | 0.57 | 4/9 (44%) | 3/8 (38%) | 5/8 (62%) |
|  | 2-step LASSO | 0.58 | 4/9 (44%) | 3/8 (38%) | 5/8 (62%) |
|  | 1 model per HOI | 0.65 | 2/9 (22%) | 2/8 (25%) | 6/8 (75%) |
|  | ICD-9 | 0.62 | 1/9 (11%) | 2/8 (25%) | 6/8 (75%) |
|  | 1-se | 0.58 | 2/9 (22%) | 2/8 (25%) | 6/8 (75%) |
|  | No screening | 0.65 | 2/9 (22%) | 2/8 (25%) | 6/8 (75%) |

**2-step LASSO**

*Description*

LASSO is a powerful automated variable selection tool that has been shown to be a promising approach for controlling complex confounding in EHR data [1, 2]. However, when the number of confounders is large, LASSO tends to be conservative by selecting too few covariates, which may inflate the false positive rate [3]. We try to better control the false positive rate by implementing an additional LASSO variable selection step [4]. In step 4C, we use a weighted LASSO linear regression of drug use against the covariates selected in Step 4A to select those covariates that predict drug status well, again with 5-fold cross validation. These additional covariates are then included in the logistic regression model in Step 5, along with the covariates selected in Step 4B.

*Results*

Results for the “2-step LASSO” method are almost identical to those for the “1-step LASSO” method. Often, very few additional covariates are selected in the second step, and the Step 4C regression model explains very little of the variance of the predictor: on average, R^2^ equals .024 in these regression models. Even when many additional covariates are selected, the regression estimates are generally little changed.

Our results demonstrate that for our data the second LASSO step in our variable selection method does not improve performance. This is likely since most of the covariates important for adjustment are already selected in the first LASSO step.

**ICD-9**

*Description*

The ICD-9 method is a modification of the “1 model per HOI” method described in the paper, in which we use ICD-9 codes instead of PHEWAS codes as comorbidities to select from as covariates. We do this to see the effect on our results of collapsing multiple ICD-9 codes into a single PHEWAS code, as described in the paper.

*Results*

Our results show that recoding ICD-9 codes using PHEWAS codes has a slightly positive effect on the results, indicating that little useful information is lost during the PHEWAS aggregation.

**1-se**

*Description*

In the paper, we described how to select an optimal LASSO threshold using 5-fold cross validation. Another threshold that is often used in practice in place of the optimal threshold is a smaller threshold, the smallest threshold whose average deviance is within 1 standard error of the optimal average deviance (“1-se threshold”). This yields a simpler model than the optimal threshold, since the threshold used is smaller and so more of the coefficients are forced to zero. The “1-se” method is a modification of the “1 model per HOI” method described in the paper in which we use the 1-se threshold in lieu of the optimal threshold.

*Results*

Results for the “1-se” method are similar to the “1 model per HOI” method using the optimal cross-validation threshold, suggesting that LASSO models fit with the optimal threshold are not overly complex.

**No screening**

*Description*

Like the LASSO threshold, another “tuning parameter” in our method is the threshold we use in Step 4A to determine which covariates to select from in our LASSO models. The “No screening” method is a modification of the “1 model per HOI” method described in the paper in which we omit Step 4A altogether and simply include all PHEWAS codes in the LASSO logistic regression of Step 4B. This allows us to determine the sensitivity of our results to this tuning parameter.

*Results*

Results for the “No screening” method are similar to the “1 model per HOI” method, indicating little sensitivity to the tuning parameter used in Step 4A.

**Pre-matching**

*Description*

Preliminary matching of cases and controls prior to regression analysis can be helpful by increasing overlap in covariate distributions between cases and controls and thereby reducing dependence on an assumption implicit in our model, *i.e.*, that each covariate has an additive effect on the log odds of the HOI, and that this additive effect does not depend on the presence or absence of any other covariates [5]. To see to what extent our analytical methods are negatively impacted by the large excess of controls over cases in our dataset, we therefore test a modification of the “1 model per HOI” method, “Pre-matching”, where, prior to variable selection, we exclude some subjects in order to create a dataset where controls and cases are better matched. We run this method multiple times, excluding more and more subjects in order to achieve better and better matching between cases and controls.

We decide which subjects to exclude from the case and control populations by using the “stratification score”, the probability that each case and control has the HOI of interest, given that subject's values of the covariates [6]. Theoretically, it is reasonable to use the stratification score to create matched case and control populations, since if there were no unmeasured confounders and we could exactly calculate the stratification score, then we could accurately estimate the causal effect of a drug on the HOI by calculating the association between them, conditional on the stratification score [7]. The stratification score is therefore a useful tool to create case and control populations with more covariate balance.

To estimate the stratification score, we predict the probability for each case and control to be a case, using the LASSO model from step 4B of our method, where we regress case-control status on demographic characteristics and comorbidities. Then we divide the range of this stratification score, [0, 1], into 10 bins, and tabulate the number of cases and controls with a stratification score in each bin. Finally, we fix a parameter *s*≥1 and exclude cases or controls by random sampling so that the ratio of the number of cases to controls in any of the bins is not greater than *s* or less than *1/s*. For example, if *s*=5 and there are 5,500 controls and 1,000 cases in the bin [0,0.1), 500 of the controls would be excluded at random from the dataset so that the ratio of controls to cases in this bin in the dataset would not exceed *s*=5. Here, we consider values of the parameter *s* between 1 and 80. For all four HOIs, *s*=80 corresponds to no cases or controls being removed from the dataset. S1 Fig shows the distribution of stratification scores before and after matching with *s*=1, *i.e.*, so that there are equal numbers of cases and controls in each of the 10 stratification score bins. S3 Table shows how matching on this stratification score makes case and control populations more similar with respect to the characteristics shown in Table 1. The populations are made more similar even with respect to characteristics that are absent from the model used to construct the stratification score, like numbers of inpatient visits and medications.

**S3 Table. Characteristics of case and control populations in Dataset 1 after stratification score matching with *s*=1.**

|  | AKI |  | ALI |  | AMI |  | GIU |  |
| --- | --- | --- | --- | --- | --- | --- | --- | --- |
|  | Control | Case | Control | Case | Control | Case | Control | Case |
| # patients | 16672 | 16672 | 6565 | 6565 | 17219 | 17219 | 4545 | 4545 |
| Mean # inpatient visits | 1.3 | 1.32 | 1.36 | 1.35 | 1.19 | 1.16 | 1.37 | 1.46 |
| Mean # outpatient visits | 0.16 | 0.21 | 0.16 | 0.2 | 0.15 | 0.11 | 0.33 | 0.16 |
| Median # medications | 45 | 38 | 38 | 28 | 28 | 32 | 28 | 39 |
| Median # ICD-9 codes | 49 | 42 | 46 | 34 | 30 | 32 | 34 | 50 |
| Median age | 67.86 | 67.47 | 56.27 | 54.98 | 66.71 | 67.3 | 58.06 | 63.24 |
| % pregnant | 0.3 | 1.7 | 2.1 | 4.3 | 1.6 | 0 | 4.2 | 0.3 |
| % age less than 1 at window end | 1 | 4.1 | 5.7 | 10.6 | 4.1 | 0.1 | 9.7 | 2.3 |

Statistics are for the 180 days ending on the index admission, so the mean number of inpatient visits includes the index admission. Ages are as of the index admission.

*Results*

We ran the “Pre-matching” method with various values of *s*, where more subjects are excluded with lower values of *s*. S2 Fig shows the AUC values for each HOI as a function of *s*. For ALI and GIU, the AUCs are highest without any exclusion of subjects (when *s*=80), and for AKI and AMI the AUCs are close to their highest values without any exclusion of subjects, suggesting that model dependence, the problem we sought to combat with pre-matching, is not a significant issue when using these models for drug-HOI odds ratio estimation.

Second dataset used for evaluation of analytical methods

**Description of Dataset 2**

The dataset presented in the paper uses admission and discharge notes. It also uses only medications from the “home medications” and the “medications on admission” sections of the notes, but not from the “medications” section of the notes. This is because it is not clear that the section heading “medications” refers to medications taken at home prior to the development of the HOI.

We construct a second dataset (which we call “Dataset 2”), where we do use the “medications” section of the notes, but for which we only use admission notes. Using only admission notes lessens the possibility that the “medications” section of the notes refers to medications prescribed at the hospital after the development of the HOI. This dataset has many more medication records than the dataset presented in the paper, and so more drugs from the OMOP reference set can be used (169 versus 94 for the dataset in the paper).

**Results for Dataset 2**

The results for Dataset 2 are presented in S4 Table. For the dataset presented in the paper, the “1 model per HOI method” led to better AUC values than the two baseline methods for all four HOIs; here AUC values are better than for the baseline methods for all HOIs except AMI, for which they are a bit worse. As for the dataset presented in the paper, the “1 model per HOI” method leads to much improved coverage probabilities under the null, but not quite reaching the nominal 95% level. The good performance of our analytical methods with this dataset, where they are tested with many more positive and negative controls, suggests that our methods are robust and may work well with other datasets also.

**S4 Table. Table of results for Dataset 2.**

| **HOI** | **Experiment type** | **AUC** | **Positive controls with one-sided p-value < 0.025** | **Negative controls with one-sided p-value < 0.025** | **Negative controls with 95% CI including null** |
| --- | --- | --- | --- | --- | --- |
| **AKI** | No adjustment, Dataset 2 | 0.65 | 10/13 (77%) | 22/32 (69%) | 9/32 (28%) |
|  | Only demographic Dataset 2 | 0.54 | 9/13 (69%) | 14/32 (44%) | 18/32 (56%) |
|  | 1 model per HOI Dataset 2 | 0.78 | 8/13 (62%) | 7/32 (22%) | 25/32 (78%) |
| **ALI** | No adjustment, Dataset 2 | 0.28 | 11/29 (38%) | 10/13 (77%) | 3/13 (23%) |
|  | Only demographic Dataset 2 | 0.31 | 5/29 (17%) | 5/13 (38%) | 8/13 (62%) |
|  | 1 model per HOI Dataset 2 | 0.37 | 1/29 (3%) | 2/13 (15%) | 10/13 (77%) |
| **AMI** | No adjustment, Dataset 2 | 0.71 | 7/16 (44%) | 6/36 (17%) | 22/36 (61%) |
|  | Only demographic Dataset 2 | 0.67 | 5/16 (31%) | 2/36 (6%) | 30/36 (83%) |
|  | 1 model per HOI Dataset 2 | 0.67 | 1/16 (6%) | 1/36 (3%) | 33/36 (92%) |
| **GIU** | No adjustment, Dataset 2 | 0.39 | 5/10 (50%) | 13/20 (65%) | 7/20 (35%) |
|  | Only demographic Dataset 2 | 0.42 | 3/10 (30%) | 8/20 (40%) | 11/20 (55%) |
|  | 1 model per HOI Dataset 2 | 0.48 | 1/10 (10%) | 3/20 (15%) | 17/20 (85%) |

**S1 Fig. Distribution of stratification scores before and after matching of cases and controls in four HOIs.**

**S2 Fig. AUC values for “Pre-matching” method as a function of *s*.**

**References**

1. Harpaz R, Haerian K, Chase HS, Friedman C. Mining electronic health records for adverse drug effects using regression based methods. Proceedings of the 1st ACM International Health Informatics Symposium; Arlington, Virginia, USA. 1883008: ACM; 2010. p. 100-7.

2. Li Y, Salmasian H, Vilar S, Chase H, Friedman C, Wei Y. A method for controlling complex confounding effects in the detection of adverse drug reactions using electronic health records. J Am Med Inform Assoc. 2014;21(2):308-14. doi: 10.1136/amiajnl-2013-001718. PubMed PMID: 23907285; PubMed Central PMCID: PMCPMC3932454.

3. Zou H, Hastie T. Regularization and variable selection via the elastic net (vol B 67, pg 301, 2005). J Roy Stat Soc B. 2005;67:768-. doi: DOI 10.1111/j.1467-9868.2005.00527.x. PubMed PMID: WOS:000233203400009.

4. Belloni A, Chernozhukov V, Wei Y. Post-Selection Inference for Generalized Linear Models with Many Controls. arXiv. 2016.

5. Gelman A, Hill J. Data analysis using regression and multilevel/hierarchical models. Cambridge ; New York: Cambridge University Press; 2007. xxii, 625 p. p.

6. Cologne JB, Shibata Y. Optimal case-control matching in practice. Epidemiology. 1995;6(3):271-5. PubMed PMID: 7619935.

7. Allen AS, Satten GA. Control for confounding in case-control studies using the stratification score, a retrospective balancing score. Am J Epidemiol. 2011;173(7):752-60. doi: 10.1093/aje/kwq406. PubMed PMID: 21402731; PubMed Central PMCID: PMCPMC3070492.
